# Assessing the Novel Myval Balloon-Expandable Valve with the Evolut Valve: A Propensity-Matched Study

**DOI:** 10.3390/jcm12134213

**Published:** 2023-06-22

**Authors:** Jonathan Halim, Maxim Rooijakkers, Peter den Heijer, Milad El Haddad, Ben van den Branden, Jeroen Vos, Bas Schölzel, Martijn Meuwissen, Menno van Gameren, Saloua El Messaoudi, Niels van Royen, Sander IJsselmuiden

**Affiliations:** 1 Department of Cardiology, Amphia Hospital Breda, Molengracht 21, 4818 CK Breda, The Netherlands; pdheijer@me.com (P.d.H.); bvandenbranden@amphia.nl (B.v.d.B.); jvos@amphia.nl (J.V.); bscholzel@amphia.nl (B.S.); mmeuwissen@amphia.nl (M.M.); mvangameren@amphia.nl (M.v.G.); sijsselmuiden@amphia.nl (S.I.); 2 Department of Cardiology, Radboud University Medical Center, Geert Grooteplein Zuid 10, 6525 GA Nijmegen, The Netherlands; max.rooijakkers@radboudumc.nl (M.R.); saloua.elmessaoudi@radboudumc.nl (S.E.M.); niels.vanroyen@radboudumc.nl (N.v.R.); 3 Department of Cardiology, AZ Sint-Jan Brugge, Ruddershove 10, 8000 Bruges, Belgium; milad.elhaddad@emdt.eu

**Keywords:** aortic valve stenosis, transcatheter aortic valve replacement, myval valve

## Abstract

Background: The Myval balloon-expandable (BE) valve has shown encouraging early clinical data in terms of safety and efficacy. Comparative data with other well-established contemporary valves are nonetheless still scarce. This study aims to compare the performance of the Myval BE valve with the Evolut self-expanding (SE) valve. Methods: In this retrospective single-center study, 223 patients with symptomatic severe aortic stenosis (AS) were included and treated with the Myval BE valve (n = 120) or with the Evolut SE valve (n = 103). Then, 91 pairs were compared after matching. Clinical outcomes were evaluated at 30 days and 1 year. Echocardiographic follow-up was performed at 30 days. Results: Procedural complications were rare in both groups. At the 30-day follow-up, no significant difference in cardiac death (Myval: 1% vs. Evolut: 2%, *p* = 0.56), stroke (2% vs. 4%, *p* = 0.41) and myocardial infarction (1% vs. 3%, *p* = 0.31) was observed. A permanent pacemaker implantation (PPI) was significantly less needed in the Myval group (4% vs. 15%, *p* = 0.01). At 1 year, cardiac death (2% vs. 4%, *p* = 0.41) and the stroke rate (7% vs. 5%, *p* = 0.76) were similar. Moderate–severe paravalvular leakage (PVL) was also comparable in both groups (1% vs. 4%, *p* = 0.17). Conclusion: Safety and efficacy outcomes were comparable between the two valves, except for a higher PPI rate for the Evolut SE valve. Up to 1-year follow-up, clinical outcomes showed acceptable rates of stroke and cardiac death with both valves. Valve hemodynamics were excellent with a low rate of moderate–severe PVL in both groups.

## 1. Introduction

Transcatheter aortic valve replacement (TAVR) has evolved rapidly and is at present indispensable for the treatment of symptomatic severe aortic stenosis (AS) [1,2,3,4,5,6,7,8]. In addition, a paradigm shift can be observed with lower-surgical-risk patients nowadays being considered for TAVR [9,10,11,12]. Nevertheless, procedure-related adverse events, such as moderate–severe paravalvular leakage (PVL) and the need for permanent pacemaker implantation (PPI), still occur and can have a negative impact on clinical outcomes. Moderate or severe PVL, for instance, has been associated with a two- to threefold higher mortality rate [13,14]. Whereas a PPI after TAVR resulting in long-term right ventricular pacing is known to have a negative impact on left ventricular remodeling and heart failure, data on its impact on mortality have so far been conflicting [15,16,17,18,19,20]. Consequently, new-generation transcatheter heart valve (THV) systems are being developed with the intent to improve clinical outcomes and to lower the risk for procedure-related adverse events.

The Evolut self-expanding (SE) valve (Medtronic, Minneapolis, MN, USA) is one of the most widely used THV systems demonstrating superior results with eminent valve performance and outstanding clinical outcomes [8]. These positive results could also be reproduced in the lower-surgical-risk group patients with symptomatic severe AS [10,11].

The Myval balloon-expandable (BE) valve (Meril Lifesciences, Vapi, India) is a novel THV system and has shown promising results, with early clinical data confirming its safety and short-term clinical efficacy [21,22,23,24]. An important feature of the Myval BE valve is the availability of a more comprehensive device size selection, compared to other BE valves, with the addition of intermediate and XL device sizes. As a result, tailored device sizing can be realized, minimizing under- or oversizing, potentially lowering the risk for procedure-related adverse events and improving clinical outcomes.

With the increased usage of the Myval BE valve worldwide, it becomes imperative to assess this novel BE valve with contemporary THV systems and address the principal differences with regard to clinical efficacy and safety. Up to now, retrospective data comparing the novel Myval BE valve and Evolut SE valve have demonstrated equivalent performance. A lower PPI and residual PVL rate, though, were seen with the Myval BE valve [24,25]. The LANDMARK trial, a randomized controlled trial, will evaluate the safety and performance of the Myval BE valve and compare them to the most established new-generation THV systems [26]. As comparative data between the Myval BE valve and Evolut SE valve are still limited, we sought to further explore the short-term safety and efficacy between these two contemporary THV systems.

## 2. Materials and Methods

### 2.1. Study Design

In this retrospective single-center study, 223 patients with symptomatic severe AS underwent TAVR with the Myval BE valve (n = 120) or the Evolut R/Pro SE valve (n = 103) between October 2019 and June 2021 in the Amphia hospital Breda, the Netherlands. In all cases, the Heart Team confirmed the indication for TAVR. Patients with a bicuspid aortic valve and a previous aortic bioprosthetic valve were excluded from this study. Informed consent was provided by all patients for the procedure and subsequent data collection for retrospective data with the study protocol conforming with the ethical guidelines of the 1975 Declaration of Helsinki. Before TAVR, a detailed preprocedural assessment was performed in each patient consisting of clinical assessment (medical history, symptoms, laboratory tests, risk evaluation and frailty risk assessment by a geriatrician), electrocardiography, echocardiography, coronary angiography and multidetector computed tomography. Device size selection and the suitability of each valve system were assessed by experienced TAVR operators by means of multidetector computed tomography using dedicated software (3mensio, Pie Medical Imaging, Maastricht, the Netherlands). The Evolut SE valve was preferred when a small aortic annulus, circumferential annular calcification or low coronary ostia was present. The choice for the Evolut SE valve when significant annular calcification was present was based on experience that, in these cases, BE valves carry a higher risk for annular rupture, since the balloon diameter in BE valves is often larger than the balloon diameter used for predilation in SE valves. In our center, the femoral artery was the default access route and was approached by surgical cutdown. The transapical (Myval BE valve) or direct aortic route (Evolut SE valve) was chosen as an alternative access site if the femoral artery was inaccessible. All patients underwent general anesthesia during TAVR, which is still considered the standard of care in our center. Predilation was left to the operator’s discretion taking into consideration the clinical and anatomical properties of the patient. After valve deployment, PVL was assessed systematically with transesophageal echocardiography. Postdilation was performed if more than mild PVL was observed. After TAVR, clinical evaluation took place at 30 days and 1 year. Transthoracic echocardiographic follow-up was carried out at 30 days. An echocardiographic assessment was performed in a core lab by two experienced imaging cardiologists who were blinded to the type of valve.

### 2.2. THV Systems

The Evolut SE valve consists of a nitinol frame with a supra-annular porcine tissue valve [27]. The Evolut Pro has an added external porcine pericardial wrap in contrast to the Evolut R. This external wrap is located at the lower end of the frame and serves to increase surface contact with the native anatomy in order to lower the risk for PVL. The Evolut R is available in 23, 26, 29 and 34 mm, while the Evolut Pro only provides 23, 26 and 29 mm valve sizes. Femoral access is obtained with a 14F/16F InLine sheath or a 20F introducer sheath depending on the valve size.

The Myval BE valve consists of a nickel-cobalt frame utilizing hexagonal cells and is constructed in a hybrid fashion [21]. The tri-leaflet valve is composed of bovine pericardium and received anti-calcification treatment (AntiCa, Meril Life Sciences, Vapi, India). In order to minimize PVL, polyethylene terephthalate was added internally and externally to the lower cells. Extensive device size selection is provided with the possibility to select intermediate valve sizes (21.5, 24.5 and 27.5 mm) and extra-large valve sizes (30.5 and 32 mm) in addition to conventional valve sizes (20, 23, 26 and 29 mm). Importantly, diameters can be further modified by adding or subtracting 1–2 mL of saline/contrast to the balloon. As a result, optimal device sizing can be achieved with 0.5 mm incremental steps in implantation diameter. All valve sizes are compatible with a 14F sheath.

### 2.3. Study Endpoints

The primary endpoint was to compare the degree of PVL, mean transvalvular gradient and aortic valve area at 30-day follow-up after matching for baseline characteristics. The PVL severity was evaluated conforming to VARC-2 criteria and classified as none, trace, mild, moderate or severe. The secondary endpoint was to compare procedural complications and clinical outcomes according to VARC-2 criteria at 30 days and 1 year. Procedural complications consisted of valve embolization, annular rupture, coronary obstruction and procedural death. Clinical outcomes at 30 days were all-cause death, cardiac death, all stroke, myocardial infarction, acute kidney injury (stage 2 or 3), new PPI and major vascular and bleeding complications. Patients were re-evaluated at 1 year with the following clinical outcomes being considered: all-cause death, cardiac death, all stroke and myocardial infarction.

### 2.4. Statistical Analysis

All continuous variables are expressed as mean and standard deviation. Categorical variables are presented as frequency and percentage. All analyses were conducted with SPSS v.26 (IBM, Chicago, IL, USA). A 2-tailed unpaired Student *t*-test was used for the comparison of continuous variables between the two groups. A chi-square test was used to evaluate the relation between two categorical variables. A two-tailed *p*-value < 0.05 was considered to be statistically significant. Data of 91 pairs were then compared after propensity score matching. Propensity scores were calculated using binary logistics for the primary composite outcome and secondary outcomes and adjusted for the following baseline characteristics: age, body mass index, Euroscore II, coronary artery disease, chronic kidney disease, cerebrovascular accident, pre-existing pacemaker, atrial fibrillation, the presence of right bundle branch block and left ventricular ejection fraction.

## 3. Results

### 3.1. Baseline Characteristics

A total of 223 patients undergoing TAVR with either the Myval BE valve (n = 120) or the Evolut SE valve (n = 103) were included in our study. The baseline characteristics of the global cohort are shown in Table 1.

Propensity score matching resulted in 91 pairs. The baseline characteristics of the matched cohort were well balanced, except for NYHA class III or IV (31% vs. 46%, *p* = 0.03), first-degree AV block (9 vs. 19%, *p* = 0.05) and moderate–severe aortic regurgitation being less prevalent in the Myval group (Table 1). In the Evolut group, a slightly higher mean aortic valve gradient (37.8 ± 13.3 vs. 43.8 ± 17.9, *p* = 0.02) could be observed as well.

### 3.2. Procedural Outcomes

The femoral artery was the predominant access site (Myval: 90% vs. Evolut: 96%, *p* = 0.15) (Table 2). Alternative access routes, such as the transapical and direct aortic approaches, were used in the remaining patients. Predilation (4% vs. 25%, *p* = 0.0001) and postdilation (3% vs. 26%, *p* ≤ 0.00001) were carried out less frequently in the Myval group. In both groups, valve embolization occurred in 1% of the patients requiring a second valve prosthesis. Moreover, a low rate of coronary obstruction (1% vs. 2%, *p* = 0.56) and procedural death (2% vs. 1%, *p* = 0.56) was observed. In the Myval group, two patients died during TAVR. In the first patient, valve deployment led to right coronary artery obstruction and a subsequent cardiac arrest. In the second patient, the valve was deployed in the abdominal aorta due to the inability of the THV to pass the severely calcified aortic valve despite predilation. Unsuccessful device retrieval forced the operator to deploy the valve in the abdominal aorta, leading to aortic rupture. In the Evolut group, one patient died during TAVR. This was caused by left main coronary artery obstruction after valve deployment. Importantly, annular rupture did not occur in our study population.

### 3.3. Clinical Outcomes

Clinical outcomes for the Myval and Evolut group are summarized in Table 2. At 30-day follow-up, no significant difference in all-cause death (3% vs. 2%, *p* = 0.65) and cardiac death (1% vs. 2%, *p* = 0.56) was present. Stroke (2% vs. 4%, *p* = 0.41), myocardial infarction (1% vs. 3%, *p* = 0.31) and acute kidney injury (5% vs. 7%, *p* = 0.76) occurred numerically less in the Myval group compared to the Evolut group, but this was not statistically significant.

**Figure 1:**
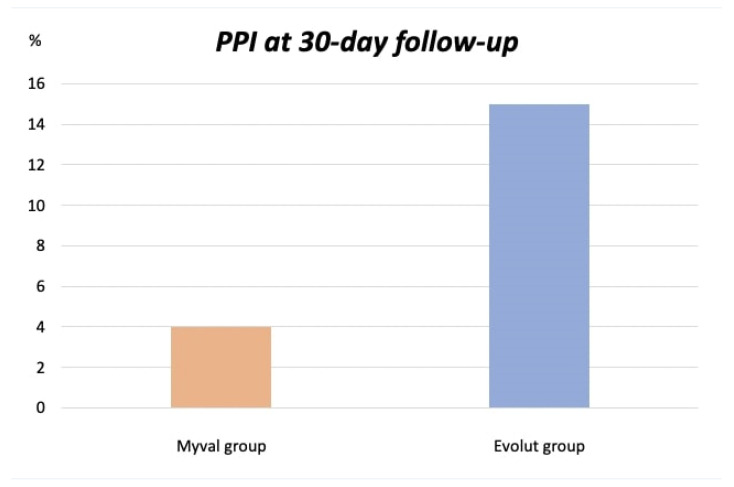
PPI rate at 30 days. Abbreviation: PPI = Permanent pacemaker implantation.

Importantly, significantly fewer patients in the Myval group developed a high-grade AV block necessitating a PPI (4% vs. 15%, *p* = 0.01) (Figure 1). Major access-site-related vascular and major bleeding complications were absent in both groups. At 1-year follow-up, clinical outcomes were comparable between the Myval and Evolut groups. The cardiac death rate was low in both groups (2% vs. 4%, *p* = 0.41). The all-cause death (9% vs. 8%, *p* = 0.79) and stroke rate were not different between groups (7% vs. 5%, *p* = 0.76).

### 3.4. Echocardiographic Outcomes at 30-Day Follow-Up

**Figure 2:**
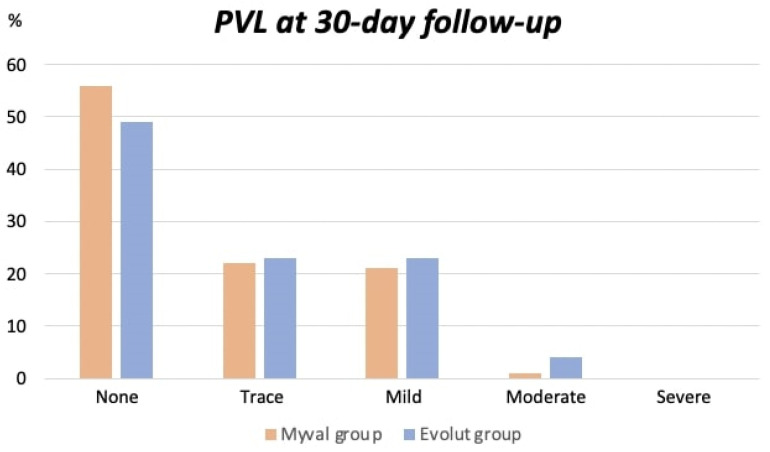
Distribution of the degree of PVL at 30 days. Abbreviation: PVL= Paravalvular leakage.

Moderate–severe PVL was observed in the Myval group in 1% of the patients in contrast to the Evolut group, in which 4% of the patients had moderate–severe PVL (*p* = 0.17) (Table 2). An overview of the distribution of PVL severity is illustrated in Figure 2.

In both groups, improved valve hemodynamics could be observed after TAVR with an increase in the aortic valve area from 0.75 ± 0.2 cm² to 1.98 ± 0.5 cm² in the Myval group and 0.78 ± 0.2 cm² to 2.13 ± 0.5 cm² in the Evolut group (*p* = 0.08).

**Figure 3:**
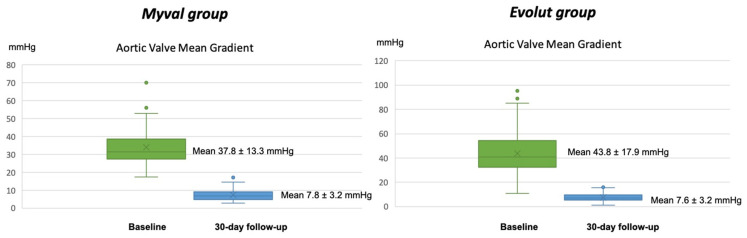
Aortic valve mean gradient at baseline compared to 30-day follow-up.

A decrease in the aortic valve mean gradient was also seen from 37.8 ± 13.3 mmHg to 7.8 ± 3.2 mmHg in the Myval group and 43.8 ± 17.9 mmHg to 7.6 ± 3.2 mmHg in the Evolut group (*p* = 0.63 for differences in delta from pre-implantation to post-implantation) (Figure 3).

## 4. Discussion

In this retrospective single-center study, the safety and performance of the novel Myval BE valve were assessed and compared with the Evolut SE valve. This is the first time these two new-generation THV systems are being compared utilizing propensity-score-matched analysis. The main findings of this study are that (1) at 1-year follow-up, the Myval BE valve is comparable to the Evolut SE valve with regard to clinical outcomes, (2) a lower PPI rate was observed with the Myval BE valve compared to the Evolut SE valve, and (3) both THV systems are associated with improved valve hemodynamics with a low rate of moderate–severe PVL.

An important detail between the two groups was the difference in the PPI rate. Previous data have indeed shown that the PPI rate of the Evolut SE valve remains relatively high compared to the Sapien 3 BE valve [7,8,9,10,11,12,28]. Moreover, a higher PPI rate with the Evolut SE valve compared to the Myval BE valve was also observed in two retrospective studies [24,25]. In our study, this finding was confirmed with a higher PPI rate in the Evolut group after correcting for the presence of an RBBB before TAVR, which is known to be a major risk factor for developing a high-grade AV block after TAVR. A lower risk for developing a high-grade AV block with the Myval BE valve could have been reinforced with the availability of intermediate valve sizes for the Myval BE valve, minimizing the risk of oversizing and contributing to a lower use of pre-and postdilation. Hence, the majority (54%) of patients in the Myval group received an intermediate valve size. It should, however, be noted that additional risk factors were not taken into account, such as the membranous septum length and implantation depth. While previous studies have shown that a PPI after TAVR can lead to an increase in heart failure hospitalizations, its impact on death has so far been conflicting [15,16,17,18,19,20]. Nevertheless, there is strong evidence from recent data that a PPI after TAVR may increase the mortality rate [29,30,31]. Interestingly, in our study, no significant difference in mortality rate was demonstrated despite the significantly lower PPI rate in the Myval group. Hence, clinical outcomes were largely comparable between the two groups and corresponded with available data from large randomized controlled trials [7,8,9,10,11,12].

Moreover, the occurrence of an acute kidney injury was relatively higher in both groups compared to previous studies. In spite of that, complete recovery of the kidney function was seen at discharge, indicating a prerenal cause of the acute kidney injury. Access-site-related major vascular and bleeding complications were absent in our study. It should be noted though that in all patients undergoing transfemoral TAVR, the femoral artery was approached surgically. In our center, surgical cutdown of the femoral artery is still being applied in most patients due to its extremely low complication rate. We acknowledge this is not considered the standard of care in most other heart centers.

Both valves were associated with excellent echocardiographic findings at 30-day follow-up. A significant improvement in the aortic valve area and mean aortic valve gradient could be observed. Moderate–severe PVL was slightly less prevalent in the Myval group compared to the Evolut group (1% vs. 4%), but this was not statistically significant. The presence of mild PVL was comparable in both groups (21% vs. 23%, *p* = 0.72). Earlier studies comparing the Sapien 3 BE valve or the Myval BE valve with the Evolut SE valve have shown superiority in terms of residual PVL when treated with a BE valve [25,32,33]. Indeed, it can be postulated that the extensive device selection provided by the Myval BE valve can minimize the risk of relative undersizing, which can lead to a lower rate of ≥mild PVL. In this study, however, the Myval BE valve did not demonstrate any superiority in residual PVL.

We can conclude that the use of the Myval BE valve is feasible and associated with good clinical outcomes at 1 year. Safety and efficacy outcomes are comparable between both valves, with a lower rate of PPI when treated with the Myval BE valve. We believe that the ability of the Myval BE valve to implant a more customized device size taking into account the patient’s anatomy can be beneficial and minimizes procedure-related adverse events, such as the need for a PPI and residual PVL. In addition, the availability of intermediate valve sizes may prevent the frequent usage of pre- and postdilation, which is a well-known risk factor for developing conduction disturbances and the subsequent need for a PPI. The LANDMARK trial will give us a definite answer on how the Myval BE valve performs compared to other well-established THV systems [26].

## 5. Limitations

The main limitation of our study is its nonrandomized design. Despite the use of propensity score matching analysis, bias from unmeasured confounders cannot be excluded. Specifically, in order to minimize the risk of annular rupture, in our center, SE valves were selected for all patients with significant annular calcification. It cannot be excluded that this selection strategy partly contributes to the higher PPI rates in the SE group. A second limitation is the relatively small sample size and a limited follow-up period of 1 year, precluding us from drawing conclusions on longer-term safety and efficacy with the Myval BE valve.

## 6. Conclusions

In this study, safety and efficacy outcomes were comparable for both valves. Up to 1 year, clinical outcomes remained excellent with a low rate of major adverse events. In both groups, favorable valve hemodynamics were observed. The use of the Myval BE valve was, however, associated with a lower rate of PPI. A large randomized trial is needed to confirm these findings.

## Figures and Tables

**Table 1:** Clinical baseline characteristics.

	Matched Cohort Myval n = 91 N (%) or Mean ± SD	Evolut n = 91 N (%) or Mean ± SD	*p*-Value	Complete Cohort Myval n = 120 N (%) or Mean ± SD	Evolut n = 103 N (%) or Mean ± SD	*p*-Value
Clinical characteristics						
Age	80.0 ± 6.1	80.5 ± 5.5	0.58	80.2 ± 6.3	80.5 ± 5.6	0.76
Male	46 (51)	46 (51)	1.00	64 (53)	53 (51)	0.78
BMI	28.3 ± 4.3	28.2 ± 4.6	0.91	28.2 ± 4.6	27.8 ± 4.6	0.49
Euroscore II	3.7 ± 3.0	3.5 ± 2.7	0.64	4.0 ± 2.8	3.5 ± 2.8	0.92
NYHA class III or IV	28 (31)	42 (46)	0.03	23 (38)	44 (43)	0.0001
Diabetes Mellitus	34 (37)	32 (35)	0.76	43 (36)	36 (35)	0.89
Hypertension	65 (71)	59 (65)	0.34	85 (71)	66 (64)	0.28
Coronary artery disease	41 (45)	37 (41)	0.55	56 (47)	45 (44)	0.66
Previous CABG	13 (14)	10 (11)	0.50	13 (11)	15 (15)	0.40
Previous valve surgery	3 (3)	2 (2)	0.65	3 (3)	3 (3)	0.85
Chronic kidney disease	31 (34)	30 (33)	0.88	42 (35)	39 (38)	0.66
Cerebrovascular disease	19 (21)	19 (21)	1.00	24 (20)	21 (20)	0.94
Peripheral vascular disease	12 (13)	12 (13)	1.00	17 (14)	12 (12)	0.58
COPD	12 (13)	17 (19)	0.31	19 (16)	18 (17)	0.74
Atrial fibrillation	26 (29)	25 (27)	0.87	39 (33)	27 (26)	0.31
Prior Pacemaker	6 (7)	5 (5)	0.76	10 (8)	5 (5)	0.30
RBBB	11 (12)	8 (9)	0.47	12 (10)	12 (12)	0.69
LBBB	7 (8)	6 (7)	0.77	11 (9)	7 (7)	0.52
1st-degree AVB	8 (9)	17 (19)	0.05	12 (10)	19 (18)	0.07
Echocardiographic measurements						
LVEF ≤ 40%	9 (10)	11 (12)	0.64	16 (13)	11 (11)	0.54
AV area, cm2	0.76 ± 0.16	0.77 ± 0.22	0.58	0.77 ± 0.18	0.77 ± 0.24	0.82
AV mean gradient, mmHg	37.8 ± 13.3	43.8 ± 17.9	0.02	37.4 ± 13.5	44.1 ± 17.6	0.003
Moderate or severe aortic regurgitation	0 (0)	14 (15)	NA	0 (0)	14 (14)	NA
Moderate or severe mitral regurgitation	14 (15)	19 (21)	0.34	18 (15)	22 (21)	0.22
MDCT measurements						
Maximum annulus diameter, mm	27.4 ± 3.7	27.1 ± 3.0	0.60	27.7 ± 3.7	27.2 ± 2.9	0.35
Minimum annulus diameter, mm	21.2 ± 2.2	21.0 ± 2.8	0.67	21.3 ± 2.2	21.1 ± 2.7	0.53
Perimeter derived diameter, mm	24.7 ± 2.1	24.5 ± 2.7	0.59	24.9 ± 2.3	24.6 ± 2.6	0.37
Area derived diameter, mm	24.1 ± 2.1	23.9 ± 2.7	0.61	24.3 ± 2.2	24.0 ± 2.6	0.37
Moderate–severe calcified aortic valve	45 (49)	56 (62)	0.10	58 (48)	66 (64)	0.02

Abbreviations: BMI = Body Mass Index, NYHA = New York Heart Association, CABG = Coronary Artery Bypass Grafting, COPD = Chronic Obstructive Pulmonary Disease, RBBB = Right Bundle Branch Block, LBBB = Left Bundle Branch Block, AVB = Atrioventricular Block, LVEF = Left Ventricular Ejection Fraction, AV = Aortic Valve, MDCT = Multidetector Computed Tomography.

**Table 2:** Procedural and clinical outcomes of the matched cohort.

	Matched Cohort Myval n = 91 N (%) or Mean ± SD	Evolut n = 91 N (%) or Mean ± SD	*p*-Value
Procedural details			
Transfemoral approach	82 (90)	87 (96)	0.15
Transapical approach	9 (10)	0 (0)	NA
Direct aortic approach	0 (0)	4 (4)	NA
Predilation	4 (4)	23 (25)	0.0001
Postdilation	3 (3)	24 (26)	<0.00001
Second valve prosthesis required	1 (1)	1 (1)	1.00
Procedural complications			
Valve embolization	1 (1)	1 (1)	1.00
Annular rupture	0 (0)	0 (0)	NA
Coronary obstruction	1 (1)	2 (2)	0.56
Procedural death	2 (2)	1 (1)	0.56
30-day outcomes			
All-cause death	3 (3)	2 (2)	0.65
Cardiac death	1 (1)	2 (2)	0.56
All stroke	2 (2)	4 (4)	0.41
Myocardial infarction	1 (1)	3 (3)	0.31
Acute kidney injury (2 or 3)	5 (5)	6 (7)	0.76
Moderate or severe paravalvular leakage	1 (1)	4 (4)	0.17
New permanent pacemaker implantation	4 (4)	14 (15)	0.01
Major vascular complications	0 (0)	0 (0)	NA
Major bleeding complications	0 (0)	0 (0)	NA
1-year outcomes			
All-cause death	8 (9)	7 (8)	0.79
Cardiac death	2 (2)	4 (4)	0.41
All stroke	6 (7)	5 (5)	0.76
Myocardial infarction	2 (2)	4 (4)	0.41

## Data Availability

The data presented in this study are available on request from the corresponding author.

## Abbreviations

Transcatheter Aortic Valve Replacement	TAVR
Aortic Stenosis	AS
Paravalvular Leakage	PVL
Permanent Pacemaker Implantation	PPI
Transcatheter Heart Valve	THV
Self-Expanding	SE
Balloon-Expandable	BE
French	F
